# Pathology of Tetanus

**Published:** 1879-06

**Authors:** 


### PATHOLOGY OF TETANUS.

In an essay on this subject in the *Transactions* of the Bristol Medico-Chirurgical Society, Dr. L. Fox states that he has found in autopsies of patients dying of tetanus (1) areas of cord softened, so as to be almost diffluent; (2) hemorrhage outside of the spinal dura mater, and gummy looking fluid beneath the arachnoid; (3) distention of the vessels and thickening of the membranes; (4) fissures in the posterior white columns; (5) softening of cord and many amyloid bodies in the gray matter; (6) creamy posterior columns, the softening seeming to be composed of colloid bodies. The colloid masses of which he speaks, and which do not take the carmine dye, are probably degenerated nuclei of the neuroglia. Dr. Fox believes that none of the lesions yet found can be considered as in any way causes of tetanus. It is more likely, he thinks, that the blood itself is in fault. In strychnine poisoning—a condition so analogous to tetanus—Dr. Harley has shown that the blood is incapable of absorbing oxygen in the usual proportion. The immediately fatal phenomena of many cases of tetanus seems to point to some such explanation. This abnormal blood imperfectly nourishes the cord, and either thus, or by its distinctly toxic effects, diminishes the resistive power of that organ—in other words, a cord so conditioned is abnormally impressible.

